# (*E*)-4-Chloro-*N*-[(5-chloro-3-methyl-1-phenyl-1*H*-pyrazol-4-yl)methyl­ene]aniline

**DOI:** 10.1107/S1600536809035727

**Published:** 2009-09-09

**Authors:** Yu-Guo Zhuang

**Affiliations:** a School of Pharmaceutical and Chemical Engineering, Taizhou University, Linhai 317000, People’s Republic of China

## Abstract

In the title compound, C_17_H_13_Cl_2_N_3_, the dihedral angle between the pyrazole ring system and 4-chloro­phenyl ring is 26.1 (2)°. The C=N bond linking the two aromatic rings has an *E* conformation.

## Related literature

For the biological properties of pyrazoles, see: Pimerova & Voronina (2001 ▶); Selvam *et al.* (2005 ▶). For the biological activity of Schiff bases, see: Rajavel *et al.* (2008 ▶); Yu *et al.* (2007 ▶). For a related stucture, see: Sun *et al.* (2007 ▶).
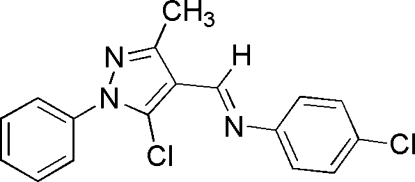


## Experimental

### 

#### Crystal data

C_17_H_13_Cl_2_N_3_
*M* *_r_* = 330.20
Orthorhombic, 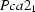

*a* = 13.6471 (6) Å
*b* = 15.6315 (3) Å
*c* = 7.3514 (6) Å
*V* = 1568.24 (15) Å^3^
*Z* = 4
Mo *K*α radiation
μ = 0.41 mm^−1^
*T* = 296 K
0.39 × 0.34 × 0.10 mm

#### Data collection

Rigaku R-AXIS RAPID diffractometer
Absorption correction: multi-scan (**ABSCOR**; Higashi, 1995 ▶) *T*_min_ = 0.839, *T*_max_ = 0.960
14704 measured reflections
3558 independent reflections
2533 reflections with *F* ^2^ > 2σ(*F* ^2^)
*R*_int_ = 0.038

#### Refinement

*R*[*F* ^2^ > 2σ(*F* ^2^)] = 0.032
*wR*(*F* ^2^) = 0.099
*S* = 1.00
3558 reflections
201 parameters
H-atom parameters constrained
Δρ_max_ = 0.21 e Å^−3^
Δρ_min_ = −0.24 e Å^−3^
Absolute structure: Flack (1983 ▶), 1627 Friedel pairs
Flack parameter: −0.02 (6)

### 

Data collection: *PROCESS-AUTO* (Rigaku, 2006 ▶); cell refinement: *PROCESS-AUTO*; data reduction: *CrystalStructure* (Rigaku/MSC, 2007 ▶); program(s) used to solve structure: *SIR97* (Altomare *et al.*, 1999 ▶); program(s) used to refine structure: *SHELXL97* (Sheldrick, 2008 ▶); molecular graphics: *ORTEP-3* (Farrugia, 1997 ▶); software used to prepare material for publication: *CrystalStructure* (Rigaku/MSC, 2007 ▶).

## Supplementary Material

Crystal structure: contains datablocks global, I. DOI: 10.1107/S1600536809035727/rk2165sup1.cif

Structure factors: contains datablocks I. DOI: 10.1107/S1600536809035727/rk2165Isup2.hkl

Additional supplementary materials:  crystallographic information; 3D view; checkCIF report

## Figures and Tables

**Fig. 1.**
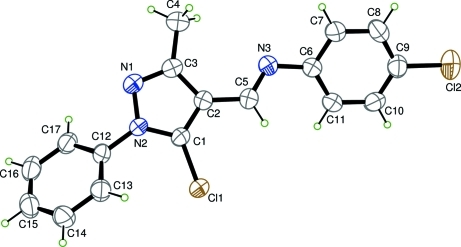
The molecular structure of title compound with the atom numbering scheme. Displacement ellipsoids are drawn at 50% probability level. H atoms are presented as a small spheres of arbitrary radius.

## supplementary crystallographic information

### Comment

Pyrazoles continue to attract a great deal of attention due to their extensive utilization in the pharmaceutical and agrochemical fields (Pimerova & Voronina, 2001; Selvam *et al.*, 2005). Schiff base compounds have been used as fine chemicals and medical substrates and they are important ligands in coordination chemistry (Rajavel *et al.*, 2008; Yu *et al.*, 2007). As part of our studies on the synthesis and characterization of pyrazoles containing Schiff base group, we report here the molecular and crystal structure of the title compound (Fig. 1).

Bond lengths and angles of the title molecule agree with those observed in a related compound, such as (*E*)-*N*-[(5-chloro-3-methyl-1-phenyl-1*H*-pyrazol-4-yl) methylene]aniline (Sun *et al.*, 2007). The dihedral angle between the pyrazole ring (N1/N2/C1–C3) and the substituted phenyl ring (C6–C11) is 26.1 (2)°. The C5═N3 bond linking the two aromatic rings has an *E*–conformation. The angles between the conjugated linkage and the pyrazole ring (C1/C2/C3/N1/N2), and between the linkage and the substituted phenyl ring (C6–C11) are 6.3 (3)° and 38.5 (2)°, respectively.

### Experimental

A solution of 5–chloro–3–methyl–1–phenyl–4–formyl–1*H*–pyrazole (5 mmol) and 4–chloroaniline (5 mmol) in ethanol (20 ml) was refluxed for 2 h. After cooling, filtration and drying, the title compound was obtained (yield: 84%, m.p. 434 K). The crystal used for data collection was obtained by slow evaporation from a saturated ethanol solution at room temperature.

### Refinement

The H atoms were positioned geometrically with C—H = 0.93Å for aromatic H and C—H = 0.96Å for methyl H and refined as riding with *U*_iso_(H) = 1.2*U*_eq_(C) and *U*_iso_(H) = 1.5*U*_eq_(methyl C).

### Crystal data

**Table:**

C_17_H_13_Cl_2_N_3_	*D*_x_ = 1.399 Mg m^−^^3^
*M**_r_* = 330.20	Melting point: 434 K
Orthorhombic, *P**c**a*2_1_	Mo *K*α radiation, λ = 0.71075 Å
Hall symbol: P 2c -2ac	Cell parameters from 10504 reflections
*a* = 13.6471 (6) Å	θ = 3.0–27.4°
*b* = 15.6315 (3) Å	µ = 0.41 mm^−^^1^
*c* = 7.3514 (6) Å	*T* = 296 K
*V* = 1568.24 (15) Å^3^	Plate, colourless
*Z* = 4	0.39 × 0.34 × 0.10 mm
*F*(000) = 680.00	

### Data collection

**Table:**

Rigaku R-AXIS RAPID diffractometer	2533 reflections with *F*^2^ > 2σ(*F*^2^)
Detector resolution: 10.00 pixels mm^-1^	*R*_int_ = 0.038
ω scans	θ_max_ = 27.4°
Absorption correction: multi-scan (*ABSCOR*; Higashi, 1995)	*h* = −17→17
*T*_min_ = 0.839, *T*_max_ = 0.960	*k* = −20→20
14704 measured reflections	*l* = −9→9
3558 independent reflections	

### Refinement

**Table:**

Refinement on *F*^2^	H-atom parameters constrained
*R*[*F*^2^ > 2σ(*F*^2^)] = 0.032	*w* = 1/[σ^2^(*F*_o_^2^) + (0.055*P*)^2^ + 0.089*P*] where *P* = (*F*_o_^2^ + 2*F*_c_^2^)/3
*wR*(*F*^2^) = 0.099	(Δ/σ)_max_ < 0.001
*S* = 1.00	Δρ_max_ = 0.21 e Å^−^^3^
3558 reflections	Δρ_min_ = −0.24 e Å^−^^3^
201 parameters	Extinction correction: *SHELXL97* (Sheldrick, 2008)
Primary atom site location: Direct	Extinction coefficient: 0.0049 (8)
Secondary atom site location: Difmap	Absolute structure: Flack (1983), 1627 Friedel pairs
Hydrogen site location: Geom	Flack parameter: −0.02 (6)

### Special details

**Table:**

Geometry. All s.u.'s (except the s.u. in the dihedral angle between two l.s. planes) are estimated using the full covariance matrix. The cell s.u.'s are taken into account individually in the estimation of s.u.'s in distances, angles and torsion angles; correlations between s.u.'s in cell parameters are only used when they are defined by crystal symmetry. An approximate (isotropic) treatment of cell s.u.'s is used for estimating s.u.'s involving l.s. planes.
Refinement. Refinement of *F*^2^ against ALL reflections. The weighted *R*–factor *wR* and goodness of fit *S* are based on *F*^2^, conventional *R*–factors *R* are based on *F*, with *F* set to zero for negative *F*^2^. The threshold expression of *F*^2^ > σ(*F*^2^) is used only for calculating *R*–factors(gt) *etc*. and is not relevant to the choice of reflections for refinement. *R*–factors based on *F*^2^ are statistically about twice as large as those based on *F*, and *R*–factors based on ALL data will be even larger.

### Fractional atomic coordinates and isotropic or equivalent isotropic displacement parameters (Å^2^)

**Table:**

	*x*	*y*	*z*	*U*_iso_*/*U*_eq_	
Cl1	0.42986 (4)	0.48323 (3)	0.33646 (10)	0.04844 (15)	
Cl2	0.32167 (6)	−0.08853 (5)	0.43233 (18)	0.0893 (3)	
N1	0.71398 (12)	0.47004 (11)	0.3343 (3)	0.0478 (4)	
N2	0.62562 (11)	0.51191 (10)	0.3340 (2)	0.0391 (3)	
N3	0.56891 (12)	0.22175 (11)	0.3631 (3)	0.0480 (5)	
C1	0.55095 (13)	0.45498 (12)	0.3464 (3)	0.0395 (4)	
C2	0.58944 (14)	0.37384 (12)	0.3577 (3)	0.0411 (4)	
C3	0.69251 (14)	0.38699 (14)	0.3490 (4)	0.0452 (5)	
C4	0.77319 (18)	0.32253 (16)	0.3538 (5)	0.0629 (7)	
C5	0.53181 (17)	0.29634 (13)	0.3730 (3)	0.0446 (5)	
C6	0.50557 (16)	0.14994 (13)	0.3773 (3)	0.0436 (5)	
C7	0.5429 (2)	0.07657 (16)	0.4568 (4)	0.0565 (6)	
C8	0.4858 (2)	0.00375 (17)	0.4763 (4)	0.0624 (7)	
C9	0.3916 (2)	0.00400 (16)	0.4118 (3)	0.0539 (6)	
C10	0.35333 (17)	0.07546 (14)	0.3280 (5)	0.0548 (6)	
C11	0.41003 (16)	0.14831 (14)	0.3120 (3)	0.0490 (6)	
C12	0.62388 (16)	0.60273 (12)	0.3108 (3)	0.0393 (5)	
C13	0.55665 (18)	0.65294 (14)	0.4025 (3)	0.0467 (5)	
C14	0.55583 (19)	0.74051 (14)	0.3718 (3)	0.0539 (6)	
C15	0.6222 (2)	0.77754 (17)	0.2553 (3)	0.0558 (7)	
C16	0.6905 (2)	0.72688 (18)	0.1672 (3)	0.0550 (6)	
C17	0.69122 (17)	0.63883 (17)	0.1930 (3)	0.0467 (5)	
H5	0.4646	0.3013	0.3909	0.054*	
H7	0.6073	0.0761	0.4978	0.068*	
H8	0.5111	−0.0447	0.5326	0.075*	
H10	0.2898	0.0746	0.2825	0.066*	
H11	0.3840	0.1968	0.2569	0.059*	
H13	0.5126	0.6284	0.4837	0.056*	
H14	0.5098	0.7746	0.4307	0.065*	
H15	0.6213	0.8363	0.2357	0.067*	
H16	0.7363	0.7520	0.0901	0.066*	
H17	0.7363	0.6047	0.1319	0.056*	
H41	0.7578	0.2762	0.2731	0.076*	
H42	0.7803	0.3010	0.4754	0.076*	
H43	0.8333	0.3490	0.3160	0.076*	

### Atomic displacement parameters (Å^2^)

**Table:**

	*U*^11^	*U*^22^	*U*^33^	*U*^12^	*U*^13^	*U*^23^
Cl1	0.0317 (2)	0.0431 (2)	0.0705 (3)	−0.0001 (2)	0.0023 (3)	0.0004 (3)
Cl2	0.0752 (5)	0.0612 (4)	0.1317 (7)	−0.0261 (3)	0.0065 (5)	0.0141 (5)
N1	0.0334 (8)	0.0459 (10)	0.0642 (12)	−0.0012 (7)	−0.0001 (12)	0.0000 (13)
N2	0.0288 (8)	0.0372 (8)	0.0513 (9)	−0.0004 (6)	0.0006 (11)	0.0029 (10)
N3	0.0417 (9)	0.0363 (9)	0.0659 (14)	−0.0003 (7)	−0.0003 (10)	0.0005 (10)
C1	0.0321 (9)	0.0388 (9)	0.0476 (11)	−0.0005 (8)	0.0036 (12)	−0.0007 (12)
C2	0.0373 (10)	0.0387 (10)	0.0473 (12)	−0.0003 (8)	0.0025 (11)	0.0031 (11)
C3	0.0367 (10)	0.0467 (11)	0.0522 (12)	0.0019 (8)	−0.0021 (12)	0.0010 (12)
C4	0.0469 (13)	0.0501 (13)	0.092 (2)	0.0085 (11)	−0.0008 (16)	0.0068 (17)
C5	0.0400 (11)	0.0424 (10)	0.0515 (15)	−0.0033 (9)	0.0034 (10)	0.0036 (11)
C6	0.0409 (11)	0.0384 (10)	0.0515 (14)	0.0015 (8)	0.0012 (10)	0.0010 (10)
C7	0.0449 (13)	0.0452 (13)	0.0795 (18)	−0.0018 (10)	−0.0114 (14)	0.0083 (13)
C8	0.0650 (19)	0.0426 (13)	0.0795 (18)	−0.0026 (11)	−0.0083 (15)	0.0170 (13)
C9	0.0514 (14)	0.0456 (12)	0.0647 (15)	−0.0067 (11)	0.0055 (13)	0.0008 (12)
C10	0.0360 (10)	0.0501 (12)	0.0782 (16)	0.0023 (10)	−0.0013 (15)	−0.0023 (14)
C11	0.0445 (13)	0.0371 (11)	0.0656 (16)	0.0045 (8)	−0.0004 (13)	0.0010 (12)
C12	0.0353 (11)	0.0362 (10)	0.0463 (13)	−0.0054 (8)	−0.0049 (10)	−0.0002 (10)
C13	0.0462 (13)	0.0439 (11)	0.0501 (13)	−0.0043 (10)	0.0019 (11)	−0.0011 (10)
C14	0.0535 (14)	0.0424 (11)	0.0658 (18)	0.0032 (10)	−0.0061 (13)	−0.0073 (13)
C15	0.0620 (18)	0.0413 (13)	0.0642 (15)	−0.0111 (12)	−0.0165 (14)	0.0050 (12)
C16	0.0493 (14)	0.0580 (16)	0.0578 (14)	−0.0171 (12)	−0.0065 (12)	0.0147 (13)
C17	0.0377 (12)	0.0513 (13)	0.0510 (13)	−0.0063 (10)	−0.0009 (10)	0.0039 (11)

### Geometric parameters (Å, °)

**Table:**

Cl1—C1	1.7121 (19)	C12—C17	1.383 (3)
Cl2—C9	1.740 (2)	C13—C14	1.387 (3)
N1—N2	1.372 (2)	C14—C15	1.375 (3)
N1—C3	1.335 (2)	C15—C16	1.384 (3)
N2—C1	1.356 (2)	C16—C17	1.389 (3)
N2—C12	1.430 (2)	C4—H41	0.960
N3—C5	1.273 (2)	C4—H42	0.960
N3—C6	1.421 (2)	C4—H43	0.960
C1—C2	1.375 (2)	C5—H5	0.930
C2—C3	1.423 (2)	C7—H7	0.930
C2—C5	1.449 (3)	C8—H8	0.930
C3—C4	1.493 (3)	C10—H10	0.930
C6—C7	1.384 (3)	C11—H11	0.930
C6—C11	1.390 (3)	C13—H13	0.930
C7—C8	1.387 (3)	C14—H14	0.930
C8—C9	1.370 (4)	C15—H15	0.930
C9—C10	1.379 (3)	C16—H16	0.930
C10—C11	1.382 (3)	C17—H17	0.930
C12—C13	1.383 (3)		
			
N2—N1—C3	105.73 (16)	C14—C15—C16	119.6 (2)
N1—N2—C1	110.32 (15)	C15—C16—C17	120.5 (2)
N1—N2—C12	119.23 (15)	C12—C17—C16	119.0 (2)
C1—N2—C12	130.32 (16)	C3—C4—H41	109.5
C5—N3—C6	118.52 (18)	C3—C4—H42	109.5
Cl1—C1—N2	123.59 (15)	C3—C4—H43	109.5
Cl1—C1—C2	127.52 (15)	H41—C4—H42	109.5
N2—C1—C2	108.80 (16)	H41—C4—H43	109.5
C1—C2—C3	103.98 (18)	H42—C4—H43	109.5
C1—C2—C5	124.65 (19)	N3—C5—H5	118.5
C3—C2—C5	131.37 (19)	C2—C5—H5	118.5
N1—C3—C2	111.16 (18)	C6—C7—H7	119.4
N1—C3—C4	119.75 (19)	C8—C7—H7	119.4
C2—C3—C4	129.1 (2)	C7—C8—H8	120.4
N3—C5—C2	123.1 (2)	C9—C8—H8	120.4
N3—C6—C7	117.5 (2)	C9—C10—H10	120.2
N3—C6—C11	124.1 (2)	C11—C10—H10	120.2
C7—C6—C11	118.4 (2)	C6—C11—H11	119.6
C6—C7—C8	121.1 (2)	C10—C11—H11	119.6
C7—C8—C9	119.3 (2)	C12—C13—H13	120.5
Cl2—C9—C8	118.8 (2)	C14—C13—H13	120.5
Cl2—C9—C10	120.3 (2)	C13—C14—H14	119.6
C8—C9—C10	120.8 (2)	C15—C14—H14	119.6
C9—C10—C11	119.6 (2)	C14—C15—H15	120.2
C6—C11—C10	120.7 (2)	C16—C15—H15	120.2
N2—C12—C13	121.07 (19)	C15—C16—H16	119.7
N2—C12—C17	117.95 (19)	C17—C16—H16	119.7
C13—C12—C17	121.0 (2)	C12—C17—H17	120.5
C12—C13—C14	119.1 (2)	C16—C17—H17	120.5
C13—C14—C15	120.8 (2)		
			
N2—N1—C3—C2	0.2 (3)	C3—C2—C5—N3	−7.3 (4)
N2—N1—C3—C4	−179.9 (2)	C5—C2—C3—N1	179.7 (2)
C3—N1—N2—C1	−0.7 (3)	C5—C2—C3—C4	−0.2 (4)
C3—N1—N2—C12	−177.0 (2)	N3—C6—C7—C8	179.8 (2)
N1—N2—C1—Cl1	−176.0 (2)	N3—C6—C11—C10	178.9 (2)
N1—N2—C1—C2	0.8 (3)	C7—C6—C11—C10	0.7 (4)
N1—N2—C12—C13	−143.5 (2)	C11—C6—C7—C8	−1.9 (4)
N1—N2—C12—C17	36.8 (3)	C6—C7—C8—C9	1.6 (4)
C1—N2—C12—C13	41.0 (3)	C7—C8—C9—Cl2	178.5 (2)
C1—N2—C12—C17	−138.7 (2)	C7—C8—C9—C10	0.1 (3)
C12—N2—C1—Cl1	−0.2 (3)	Cl2—C9—C10—C11	−179.7 (2)
C12—N2—C1—C2	176.6 (2)	C8—C9—C10—C11	−1.3 (4)
C5—N3—C6—C7	−147.9 (2)	C9—C10—C11—C6	0.9 (4)
C5—N3—C6—C11	33.9 (3)	N2—C12—C13—C14	−178.2 (2)
C6—N3—C5—C2	−179.1 (2)	N2—C12—C17—C16	179.6 (2)
Cl1—C1—C2—C3	176.1 (2)	C13—C12—C17—C16	−0.1 (3)
Cl1—C1—C2—C5	−3.4 (4)	C17—C12—C13—C14	1.5 (3)
N2—C1—C2—C3	−0.6 (3)	C12—C13—C14—C15	−1.6 (3)
N2—C1—C2—C5	179.87 (19)	C13—C14—C15—C16	0.2 (4)
C1—C2—C3—N1	0.2 (3)	C14—C15—C16—C17	1.2 (4)
C1—C2—C3—C4	−179.6 (3)	C15—C16—C17—C12	−1.3 (3)
C1—C2—C5—N3	172.0 (2)		
